# Ontogenetic Variation in Multidimensional Leaf Traits and Stage-Specific Soil–Trait Associations in *Fraxinus mandshurica*

**DOI:** 10.3390/plants15203155

**Published:** 2026-10-15

**Authors:** Mingyang Cao, Mingyuan Jin, Qingxin Mao, Jian Li, Zhengming Ji, Yunlong Wang, Yunfei Diao, Yuwei Sun, Mingyue Jin, Honghui Teng

**Affiliations:** 1 College of Engineering, Jilin Normal University, Siping 136000, China; 2 School of Economics and Management, Harbin Huade University, Harbin 150025, China; 3 College of Life Sciences, Jilin Normal University, Siping 136000, China; 4 Heilongjiang Institute of Ecology, Harbin 150081, China

**Keywords:** intraspecific trait variation, leaf functional strategies, multidimensional traits, ontogeny, soil–trait associations

## Abstract

Multidimensional trait frameworks are increasingly used to understand how plant functional strategies change across ontogeny. However, whether leaf economic, stomatal, and non-structural carbohydrate traits show consistent or dimension-specific ontogenetic patterns, and how their associations with soil factors vary across ontogenetic stages, remains unclear. We measured 11 leaf functional traits and five soil factors across sapling, young, and adult tree stages of *Fraxinus mandshurica* in a temperate mixed broadleaf–Korean pine forest. Principal component analysis was used to integrate the measured traits into leaf economic, stomatal, and non-structural carbohydrate axes, whose ontogenetic patterns and stage-specific associations with soil factors were subsequently evaluated. The results showed that the dominant structure of trait covariation shifted from a leaf economic dimension in saplings and young trees to a stomatal size–density dimension in adults. The three functional axes showed distinct stage-specific patterns: scores on the leaf economic axis decreased, and those on the stomatal axis increased from saplings to young trees, with neither differing significantly between young and adult trees, whereas scores on the non-structural carbohydrate axis did not differ significantly between saplings and young trees but were higher in adults. Stage-specific analyses revealed contrasting patterns of soil–trait associations among ontogenetic stages and functional axes, with a broader set of significant associations in saplings and young trees than in adults. Notably, soil water content was the only soil factor associated with all three functional axes in saplings, whereas soil total phosphorus made the largest independent contribution to all three axes in young trees; in adults, only scores on the leaf economic axis were significantly associated with soil pH. Our findings highlight that leaf functional variation is multidimensional, with leaf economic, stomatal, and non-structural carbohydrate dimensions exhibiting distinct ontogenetic patterns, and that soil–trait associations vary among ontogenetic stages and functional axes. These results improve our understanding of variations in multidimensional leaf functional strategies and soil–trait associations across ontogenetic stages.

## 1. Introduction

Integrating multiple trait dimensions provides a more comprehensive understanding of plant functional strategies across ontogeny than single-trait approaches [1,2,3]. The leaf economics spectrum describes coordinated variation in leaf construction costs, nutrient investment, and resource returns, ranging from acquisitive leaves with low construction costs and rapid returns to conservative leaves with greater structural investment and slower returns [4,5]. However, stomatal anatomy and leaf non-structural carbohydrate status represent aspects of leaf function not fully described by this axis: stomatal size and stomatal density jointly characterize the anatomical potential for CO_2_ diffusion and water exchange, whereas leaf soluble sugars and starch constitute major non-structural carbohydrate pools that support metabolism and help buffer imbalances between carbon supply and demand, with soluble sugars also contributing to osmotic regulation [6,7]. Integrating leaf economic, stomatal, and non-structural carbohydrate dimensions within a common multidimensional trait framework may therefore improve our understanding of plant functional strategies and their variations across ontogeny.

Tree ontogeny involves changes in tree height, canopy light environment, root-to-leaf hydraulic path length, and resource allocation among growth, maintenance, and reproduction [8,9,10]. These changes may differentially constrain distinct dimensions of leaf function. Leaf economic traits may shift towards greater resource conservation with increasing tree size, although the direction and magnitude of ontogenetic change vary among traits and species [11,12]. Stomatal traits are associated with both light and hydraulic conditions: higher irradiance is often linked to greater potential CO_2_ diffusional capacity, whereas longer hydraulic pathways in taller trees may increase resistance to water transport [13,14]. Their ontogenetic patterns may therefore differ from those of leaf economic traits. Non-structural carbohydrates may reflect the balance between photosynthetic carbon supply and sink demand, and their ontogenetic variations may indicate changes in carbon source–sink relationships [2,15]. However, whether leaf economic, stomatal, and non-structural carbohydrate traits exhibit consistent or dimension-specific ontogenetic patterns has rarely been evaluated.

Associations between leaf traits and local soil factors may differ among functional dimensions [16,17]. Soil water content (SWC) is closely linked to leaf water status, stomatal traits, and transpiration, whereas soil pH and total carbon, nitrogen, and phosphorus content (STC, STN, and STP) may covary with leaf construction, photosynthetic capacity, and non-structural carbohydrate status [18,19,20]. Moreover, as trees grow, the spatial extent of their root systems and the scale of resource integration may increase, potentially weakening the correspondence between leaf traits and local soil factors [14,21]. For example, among nine tropical Inga species, the association between leaf nitrogen content and soil-type preference differed between saplings and adults, whereas corresponding associations for several other leaf traits did not differ between stages [22]. However, soil–trait associations have rarely been compared across ontogenetic stages for multiple leaf trait dimensions, and it remains unclear whether the relative explanatory contributions of individual soil factors differ among dimensions.

We studied *Fraxinus mandshurica*, a dominant deciduous broadleaf tree in temperate mixed broadleaf–Korean pine (*Pinus koraiensis*) forests of northeastern China. We compared leaf economic, stomatal, and non-structural carbohydrate traits among saplings, young trees, and adult trees and evaluated their associations with soil factors. We tested two hypotheses: (H1) The three trait dimensions would exhibit different ontogenetic patterns: saplings would show a more acquisitive leaf economic strategy than young and adult trees, adult trees would have higher leaf non-structural carbohydrate concentrations than saplings and young trees, and the stomatal dimension would show a stage-specific pattern distinct from those of the leaf economic and non-structural carbohydrate dimensions. (H2) Soil–leaf trait associations would vary with both ontogenetic stage and trait dimension. Because adult trees may integrate soil resources over a broader spatial scale, we expected weaker associations with soil factors in adult trees than in saplings and young trees. We also expected the relative explanatory contributions of individual soil factors to differ among trait dimensions. To test these hypotheses, we measured 11 leaf traits and five soil factors, derived composite axes for the three trait dimensions, and quantified the ontogenetic variation in axis scores and their stage-specific associations with soil factors.

## 2. Materials and Methods

### 2.1. Study Site

This study was conducted in the Muling *Taxus cuspidata* National Nature Reserve, Heilongjiang Province, northeastern China (130°00′–130°28′ E, 43°49′–44°06′ N). The sampling area was located within a 25 ha permanent forest dynamics plot in a well-preserved section of the reserve that has not been subject to direct forest management since 1980 [23]. The study area is characterized by a temperate mixed broadleaved–Korean pine forest, with elevations ranging from 658 to 781 m above sea level. The predominant soil group at the study site is Cambisols according to the World Reference Base for Soil Resources (WRB) classification [24]. Soils in the Muling reserve are generally shallow, approximately 20–40 cm deep, with a thin dark surface layer and a compact brown subsoil of variable thickness [25]. The region has a northern temperate continental monsoon climate, with a mean annual temperature of approximately 2.8 °C and annual precipitation of about 530 mm. Within the same 25 ha permanent plot, Fraxinus mandshurica had an abundance of 961 individuals and an importance value of 2.79% [25].

### 2.2. Sample Collection

Field sampling was conducted in July–August 2024 during the peak growing season in the Muling Taxus cuspidata National Nature Reserve. Three size-defined ontogenetic stages of Fraxinus mandshurica—saplings, young trees, and adult trees—were distinguished based on diameter at breast height and tree height, with 10 individual trees sampled per stage. All sampled trees occurred on the same slope aspect and at similar slope angles. To limit potential spatial dependence among samples, a minimum distance of 10 m was maintained between neighbouring trees. Within the south-facing upper canopy of each tree, a trained climber randomly selected one branch [26]. Saplings had a mean diameter at breast height (DBH) of 4.72 ± 0.21 cm and a mean height of 7.06 ± 0.25 m, young trees had a mean DBH of 15.81 ± 0.63 cm and a mean height of 18.84 ± 0.36 m, and adult trees had a mean DBH of 21.92 ± 2.43 cm and a mean height of 33.83 ± 3.49 m. DBH and tree height are reported as means ± standard deviations (SD). To characterize the local canopy structure at the sampling locations, three hemispherical photographs were taken for each tree using a Nikon Coolpix 4500 digital camera fitted with a 180° fisheye lens (Nikon Corp., Tokyo, Japan). The images were analyzed using Gap Light Analyzer version 2.0 to calculate canopy openness, and the three estimates were averaged to obtain one tree-level value. Mean canopy openness was 1.83 ± 0.86% for saplings, 2.12 ± 0.55% for young trees, and 1.55 ± 0.51% for adult trees (mean ± SD; n = 10 individuals per stage).

For each tree, 15–20 fully expanded and undamaged compound leaves were collected from the selected south-facing branch in the upper canopy. Leaflet positions within each compound leaf were numbered sequentially from the apex to the base along the rachis. At each paired position, the left and right leaflets were treated as technical replicates, and their measurements were averaged following Jin et al. [27]. Measurements obtained from the sampled compound leaves were subsequently averaged within each position for each tree, yielding one tree-level mean for each of the six leaflet positions. Thus, each tree contributed six position-specific leaflet observations to the subsequent analyses. Because leaflets beyond position 6 were frequently damaged, only positions 1–6 were retained. Fresh leaflets were transported immediately to the laboratory, and measurements requiring fresh material were completed within 6 h of collection. Leaflets intended for stomatal analysis were fixed in FAA solution containing 70% ethanol, formalin, and glacial acetic acid (90:5:5 ratio), whereas additional samples were oven-dried for the determination of leaf nitrogen and non-structural carbohydrate concentrations.

Before soil sampling, surface litter was removed within a 1 m radius around each tree. Soil samples were then collected from the 0–10 cm soil layer at three equidistant points spaced 120° apart around each tree [28]. The three subsamples were thoroughly homogenized and combined into a single composite sample for subsequent soil factor measurements.

### 2.3. Leaf Trait Measurements

The leaf economic traits measured included the specific leaf area, leaf dry matter content, leaf thickness, and leaf nitrogen content. Leaf thickness was measured at the midpoint of each leaflet using a digital micrometre, avoiding the major veins. Fresh leaflets were then weighed and scanned, and their projected areas were quantified using an image analysis system. The samples were subsequently oven-dried at 65 °C to constant mass and weighed again to determine dry mass. Specific leaf area was expressed as leaf area per unit dry mass, whereas leaf dry matter content was expressed as dry mass per unit fresh mass. The oven-dried samples were finely ground, and leaf nitrogen content was determined using a K9840 Kjeldahl nitrogen analyser (Hanon, Jinan, China).

Stomatal traits included the stomatal density, stomatal length, stomatal width, and stomatal pore index. For each tree and leaflet position, three leaflets were randomly selected from the FAA-preserved material and air-dried. Clear nail varnish was applied to an approximately 1 mm^2^ vein-free area in the central region of the abaxial surface. After drying, the impressions were removed, mounted on glass slides, and observed under a light microscope with a 20× objective lens. Three non-overlapping fields were randomly imaged from each impression [29]. Stomatal density was calculated as the number of stomata per unit image area (stomata mm^−2^), while stomatal length and width were measured for three randomly selected stomata per field using ImageJ version 1.54f. Measurements were averaged across fields and the three replicate leaflets to obtain a mean for each leaflet position within each tree. The stomatal pore index (%) was calculated as (stomatal length)^2^ × stomatal density × 10^−4^, with stomatal length expressed in μm and stomatal density in mm^−2^.

Leaf soluble sugars and starch were quantified using a modified phenol–sulphuric acid method [30]. A sucrose stock solution was prepared by drying analytical-grade sucrose at 80 °C to constant mass, dissolving 0.1000 g in distilled water, and bringing the solution to 100 mL; working standards were prepared by serial dilution. For soluble sugar extraction, approximately 40 mg of ground leaf material was mixed with 10 mL of 80% ethanol for 24 h and centrifuged at 4000 rpm for 10 min. The remaining pellet was re-extracted with 5 mL of 80% ethanol and centrifuged at 4000 rpm for 5 min. The supernatants were combined and brought to 100 mL with 80% ethanol. For leaf starch extraction, the ethanol-insoluble residue was dried, suspended in 10 mL of distilled water, and gelatinised in a water bath for 15 min. After cooling to 60 °C, 1 mL of 0.5% amylase was added, followed by incubation at 60 °C for 1 h and enzyme inactivation in a boiling water bath. The hydrolysate was centrifuged at 2000 rpm for 5 min, filtered, and brought to 50 mL with distilled water. For colorimetric determination, 1 mL of each extract was mixed with 1 mL of 28% phenol, prepared in 80% ethanol, and 5 mL of concentrated sulphuric acid, shaken for 1 min, and allowed to stand for 15 min. Absorbance was measured at 490 nm using a T6 New Century UV–visible spectrophotometer (Beijing Purkinje General Instrument Co., Ltd., Beijing, China). Leaf soluble sugar and starch concentrations were calculated from the sucrose calibration curve and expressed as percentages of leaf dry mass (%). The total non-structural carbohydrate concentration was calculated as the sum of soluble sugar and starch concentrations.

### 2.4. Soil Factor Measurements

SWC was determined from fresh soil subsamples by recording their mass before and after oven-drying and was expressed as the mass of water per unit oven-dry soil mass. Soil pH was measured in a soil–water suspension using an HI 2211 benchtop pH metre (Hanna Instruments, Woonsocket, RI, USA). Dried and finely ground soil samples were analyzed for STC by dry combustion using a vario MAX CN elemental analyser (Elementar, Hanau, Germany). STN was determined by the Kjeldahl method using a K9840 Kjeldahl nitrogen analyser (Hanon, Jinan, China), and STP was determined colorimetrically following acid digestion. Definitions and units of the measured soil factors are summarized in Table S1.

### 2.5. Statistical Analyses

All statistical analyses were performed in R (version 4.2.1). Differences in individual leaf traits and soil factors among ontogenetic stages were tested using one-way analyses of variance followed by Fisher’s least significant difference tests.

Two complementary principal component analysis (PCA) approaches were used. First, PCAs based on all leaf traits were conducted separately for each ontogenetic stage and for all trees pooled to characterize multidimensional trait variation and stage-specific loading patterns. Second, pooled PCAs were conducted separately for the leaf economic, stomatal, and non-structural carbohydrate trait domains to derive axes comparable among stages. All traits were centred and scaled to unit variance before analysis. The first principal component of each domain was retained as PC_eco_, PC_sto_, and PC_NSC_, respectively, and the pooled PCA loadings were used to calculate the scores for all leaflet observations.

Leaflet-level PC scores were analyzed using linear mixed-effects models, with ontogenetic stage, leaflet position, and their interaction as fixed effects, and tree identity as a random intercept to account for repeated observations within trees. Fixed effects were evaluated using likelihood ratio tests, followed by Tukey-adjusted pairwise comparisons when stage effects were significant.

For soil–trait analyses, each tree-level soil measurement was matched to its six position-specific PC_eco_, PC_sto_, and PC_NSC_ scores. Separate multiple linear models were fitted for each ontogenetic stage and functional axis, with PC scores as response variables and SWC, pH, STC, STN, and STP as predictors. All variables were standardized before analysis. Standardized regression coefficients (β), 95% confidence intervals, and *p*-values were reported, and multicollinearity was assessed using variance inflation factors. Hierarchical partitioning was used to quantify the independent contribution of each soil factor to the full-model R^2^, expressed as percentage points of the total response variance.

## 3. Results

### 3.1. Ontogenetic Variations in Leaf Functional Trait Axes

Descriptive statistics of individual leaf traits across ontogenetic stages are presented in Table 1. PCAs based on all 11 leaf traits revealed a shift in the dominant dimension of trait covariation from leaf economics in saplings and young trees to stomatal size–density variations in adults. PC2 was dominated by non-structural carbohydrate and starch concentrations across stages. The first three components explained 58.9–69.0% of the variation within stages and 66.0% in the pooled analysis (Figure S1, Table 2).

Separate pooled PCAs for the three trait domains yielded common functional axes (Figure S2). Higher PC_eco_ scores indicated a more acquisitive leaf economic strategy. Higher PC_sto_ scores indicated larger stomata, a higher stomatal pore index, and lower stomatal density, whereas higher PC_NSC_ scores indicated greater soluble sugar, starch, and total non-structural carbohydrate concentrations.

**Figure 1:**
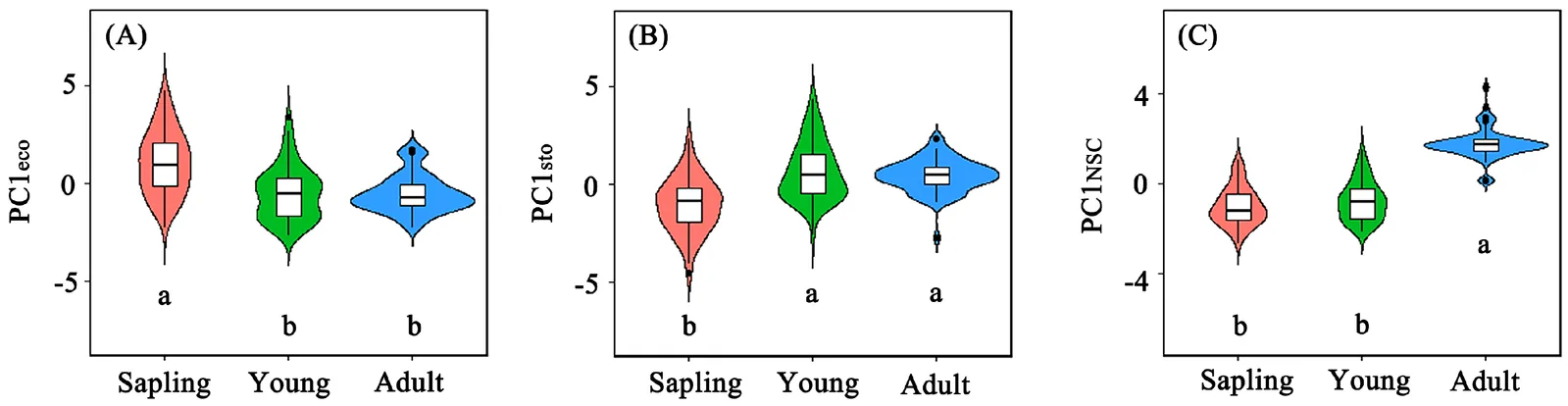
Ontogenetic variation in scores along the (**A**) leaf economic (PC_eco_), (**B**) stomatal (PC_sto_), and (**C**) non-structural carbohydrate (PC_NSC_) axes of *Fraxinus mandshurica*. Violin plots show score distributions across sapling, young tree, and adult tree stages, with embedded boxplots indicating medians and interquartile ranges. Different lowercase letters indicate significant differences among ontogenetic stages (*p* < 0.05).

Scores on all three functional axes differed significantly among ontogenetic stages (all *p* < 0.001; Figure 1, Table S2). Saplings had higher PC_eco_ and lower PC_sto_ scores than young and adult trees, which did not differ significantly in either axis. Adult trees had higher PC_NSC_ scores than saplings and young trees, which did not differ significantly. Leaflet position significantly affected PC_eco_ and PC_NSC_, but not PC_sto_, and no significant stage × leaflet position interaction was detected for any axis (Table S2).

### 3.2. Stage-Specific Associations Between Soil Factors and Leaf Functional Axes

**Figure 2:**
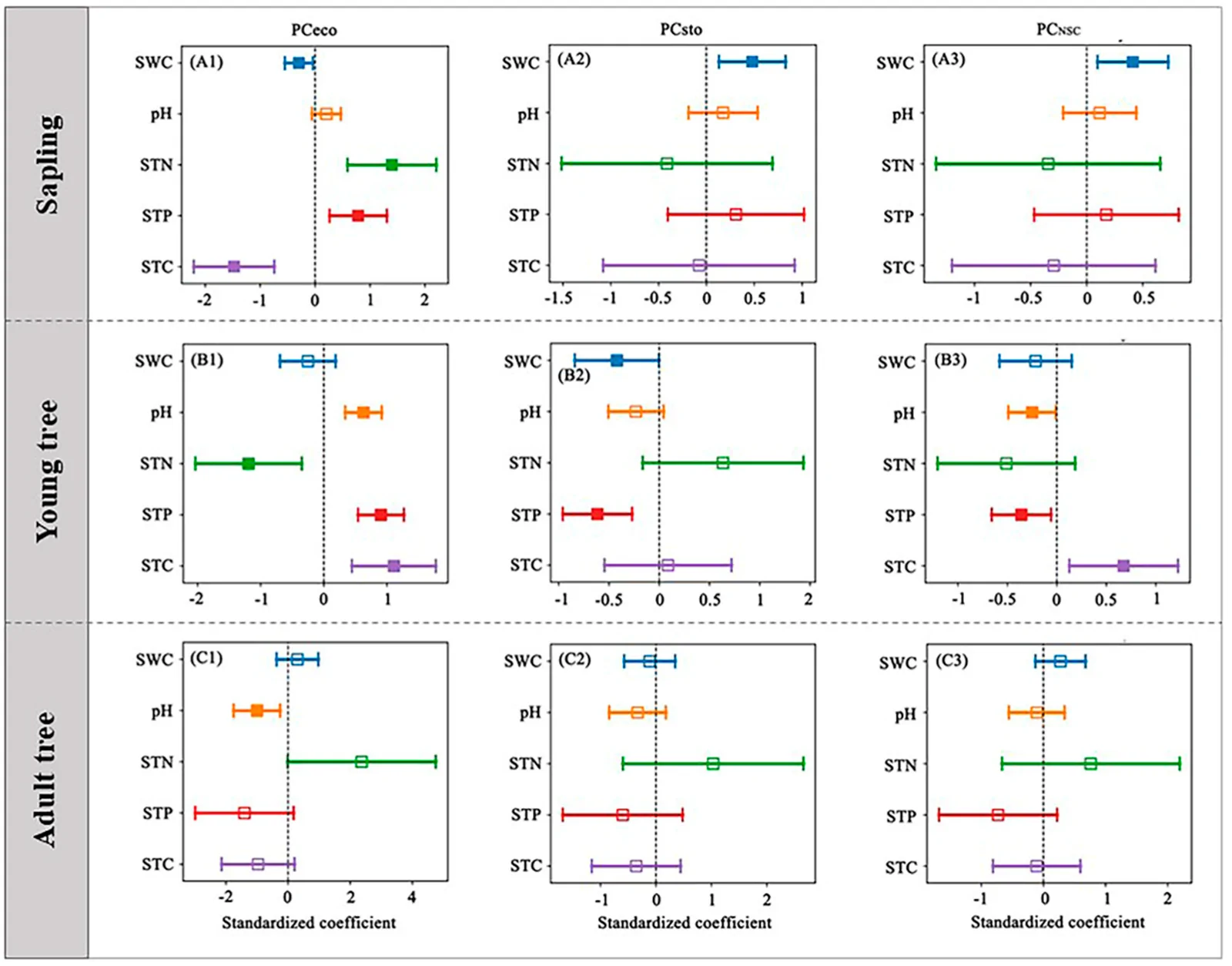
Standardized regression coefficients from stage-specific multiple linear models relating soil factors to the leaf economic (PC_eco_), stomatal (PC_sto_), and non-structural carbohydrate (PC_NSC_) axes of *Fraxinus mandshurica*. Models were fitted using position-specific PC scores, with the soil measurements for each tree matched to its six leaflet-position observations. Panels (**A1**–**A3**), (**B1**–**B3**), and (**C1**–**C3**) represent saplings, young trees, and adult trees, respectively; panels 1–3 correspond to PC_eco_, PC_sto_, and PC_NSC_. Squares indicate coefficient estimates, and horizontal lines represent 95% confidence intervals. Filled and open squares denote significant and non-significant associations, respectively (*p* < 0.05). The vertical dashed line indicates zero. SWC, soil water content; STC, soil total carbon; STN, soil total nitrogen; STP, soil total phosphorus.

**Figure 3:**
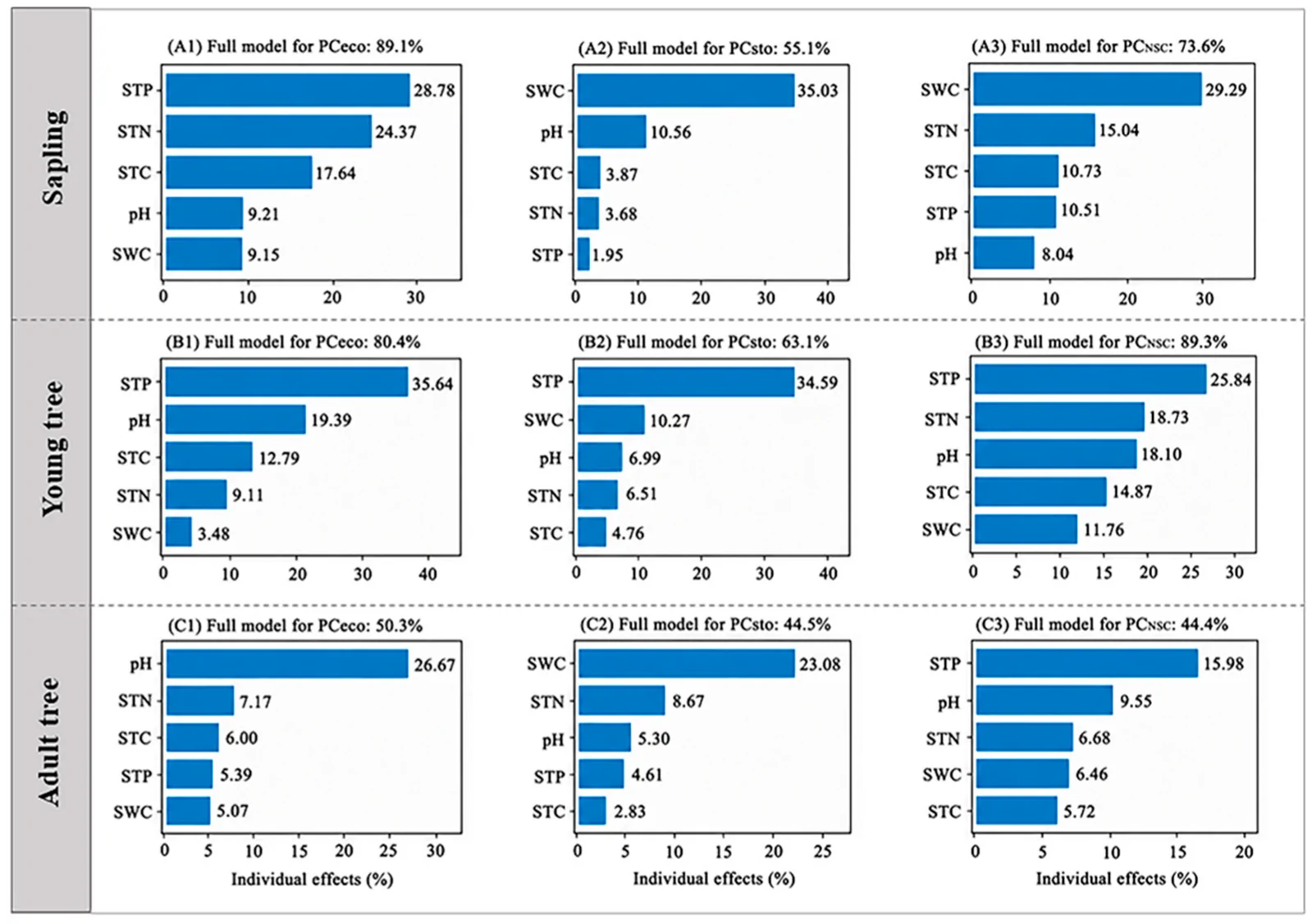
Independent contributions of soil factors to variation in the leaf economic (PC_eco_), stomatal (PC_sto_), and non-structural carbohydrate (PC_NSC_) axes of Fraxinus mandshurica, quantified by hierarchical partitioning of stage-specific multiple linear models. Models were fitted using position-specific PC scores, with the soil measurements for each tree matched to its six leaflet-position observations. Panels (**A1**–**A3)**, (**B1**–**B3**), and (**C1**–**C3**) represent saplings, young trees, and adult trees, respectively; panels 1–3 correspond to PC_eco_, PC_sto_, and PC_NSC_. Panel titles report the full-model R^2^ (%), and bars indicate the percentage points of the total response variance independently explained by each soil factor; consequently, the bar values within each panel sum to the corresponding full-model R^2^. SWC, soil water content; STC, soil total carbon; STN, soil total nitrogen; STP, soil total phosphorus.

The proportion of variation explained by the full soil models varied in magnitude among ontogenetic stages and functional axes (Figure 2 and Figure 3, Table S3). Full-model R^2^ ranged from 55.1% to 89.1% in saplings, from 63.1% to 89.3% in young trees, and from 44.4% to 50.3% in adult trees (Figure 3).

In saplings, PC_eco_ was negatively associated with SWC and STC and positively associated with STN and STP, whereas PC_sto_ and PC_NSC_ were positively associated only with SWC. STP and STN made the largest independent contributions to PCeco. In young trees, PC_eco_ was positively associated with pH, STP, and STC and negatively associated with STN. PC_sto_ was negatively associated with SWC and STP, whereas PC_NSC_ was negatively associated with pH and STP and positively associated with STC. STP made the largest independent contribution to all three axes. In adult trees, the only significant soil–axis association was a negative relationship between pH and PC_eco_ (Figure 2 and Figure 3).

## 4. Discussion

### 4.1. Dimension-Specific Ontogenetic Shifts in Leaf Functional Strategies

Within-stage PCAs revealed a multidimensional structure of leaf trait variation that shifted with ontogeny, with leaf economic, stomatal, and non-structural carbohydrate traits contributing differently to the major axes across stages. Notably, the dominant structure of trait covariation shifted from a primarily leaf economic dimension in saplings and young trees to a stomatal size–density dimension in adults (Figure S1, Table 2). This is consistent with growing evidence that plant functional variation is multidimensional and cannot be fully captured by the leaf economics spectrum [9,31,32]. Scores along the three common functional axes (PC_eco_, PC_sto_, and PC_NSC_) differed significantly among ontogenetic stages but followed contrasting stage-specific patterns (Figure 1). These stage-associated differences may, however, reflect the combined effects of developmental changes, plastic acclimation to variations in irradiance, vapour pressure deficit, and wind exposure among canopy positions, and increasing hydraulic constraints with tree height [16,33]. Although leaf sampling was standardized to south-facing branches in the upper canopy of each tree, neither canopy microclimate nor plant hydraulic traits were measured directly, preventing us from determining the relative contributions of these mechanisms. Future studies integrating longitudinal observations with direct measurements of canopy microclimate and hydraulic traits would help distinguish developmental effects from environmental acclimation. Overall, our results support H1 with respect to the observed stage-associated patterns, although the mechanisms underlying these patterns require further testing. Our results show that leaf functional dimensions vary differently among ontogenetic stages rather than following a uniform shift along a single acquisitive–conservative continuum.

The higher PC_eco_ scores observed in saplings reflected their higher specific leaf area and lower leaf dry matter content and thickness, indicating lower structural investment and a more resource-acquisitive strategy (Figure 1A). Similar ontogenetic patterns have been reported in other woody plants, with adult trees generally exhibiting higher leaf mass per area and thicker leaves than earlier ontogenetic stages [11,13]. However, PC_eco_ did not differ between young and adult trees (Figure 1A), indicating that the major shift in PC_eco_ occurred between the sapling and young tree stages rather than progressing continuously towards the conservative end of the spectrum.

Stomatal anatomy underpins leaf gas exchange and water regulation and is jointly constrained by light and hydraulic conditions [34,35]. PC_sto_ increased significantly from saplings to young trees and then remained stable in adults, largely reflecting an increase in stomatal size (Figure 1B, Table 1). Smaller stomata can respond more rapidly to short-term fluctuations in irradiance and vapour pressure deficit, which may be advantageous under the heterogeneous light conditions experienced by saplings [34]. As trees grow, changes in light exposure and hydraulic demand may favour different stomatal configurations [10,35]. Similar ontogenetic differences have been reported in Mediterranean oaks, where adults had larger stomata and a higher stomatal pore index than younger individuals [29]. Thus, ontogenetic variations in PC_sto_ may reflect stomatal anatomical adjustments to changing light and hydraulic constraints, thereby contributing to the coordination of carbon gain and water use as trees increase in size.

PC_NSC_ was higher in adult trees than in saplings and young trees, largely reflecting higher soluble sugar and starch concentrations (Figure 1C, Table 1). Non-structural carbohydrates constitute a dynamic carbon pool that buffers imbalances between photosynthetic carbon supply and demands for growth, respiration, and tissue maintenance [36,37]. The higher PC_NSC_ in adults may therefore indicate a lower relative sink demand and greater accumulation of labile carbon at later ontogenetic stages, consistent with the growth–storage trade-off reported in woody plants [2,38,39]. Notably, elevated non-structural carbohydrate concentrations cannot distinguish active storage from reduced carbon utilization [40]. Moreover, PC_eco_ and PC_sto_ did not differ between young and adult trees, whereas PC_NSC_ was higher in adults than in both earlier stages, suggesting that carbon source–sink balance may continue to shift during later ontogeny even after major changes in leaf economic and stomatal traits have stabilized.

### 4.2. Ontogenetic Shifts in Soil–Trait Associations Across Functional Dimensions

Consistent with our second hypothesis, soil–trait associations in Fraxinus mandshurica varied across ontogenetic stages and among leaf functional dimensions. Soil–trait associations were more extensive in saplings and young trees but were largely non-significant in adult trees, except for the PC_eco_–pH relationship, while the relative importance of soil factors differed among ontogenetic stages and functional dimensions (Figure 2 and Figure 3). This pattern is consistent with evidence that trait–environment relationships vary with ontogeny as tree size and resource-use strategies change during development [16,41]. Such ontogenetic variations may further reflect changes in rooting extent and the spatial scale of below-ground resource acquisition, which alter how trees access and integrate soil water and nutrients as they increase in size [42,43,44].

In saplings, soil–trait associations were strongly dimension dependent. SWC was the only soil factor significantly associated with all three functional axes (PC_eco_, PC_sto_, and PC_NSC_; Figure 2A1–A3). This pattern may be related to ontogenetic differences in below-ground water acquisition, as smaller individuals tend to rely more strongly on shallower soil water than larger trees [44]. PC_eco_ was also positively associated with STN and STP and negatively associated with STC, with STP and STN showing the largest independent contributions (Figure 2 and Figure 3). Similar associations between leaf economic traits and soil factors have been reported for temperate broadleaved trees [45]. Pierick et al. [46] likewise found that an above- and below-ground acquisition–conservation axis was associated with local soil N availability, but not P availability: this contrast may be related to differences in nutrient limitation and the P pools quantified across studies. By contrast, neither PC_sto_ nor PC_NSC_ was associated with soil factors other than SWC, which is consistent with the close linkage of stomatal anatomy to plant water relations and of non-structural carbohydrate concentrations to carbon source–sink balance [47,48].

We found that, in young trees, SWC was significantly associated only with PC_sto_, contrasting with its cross-dimensional associations in saplings (Figure 2). This shift may be related to the broader spatial scale of below-ground water acquisition as tree size increases, which may weaken the linkage between leaf function and local SWC [49,50]. PC_eco_ remained significantly associated with multiple soil factors, indicating that the leaf economic strategy remained linked to local soil nutrient conditions at the young tree stage (Figure 2 and Figure 3). Notably, the consistently high independent contribution of STP across all three axes may reflect the increasing importance of P-dependent processes during rapid growth: P supports leaf construction through RNA and phospholipid synthesis [51], may shape stomatal development through epidermal cell division and expansion [52], and is central to ATP-dependent carbon assimilation and carbohydrate partitioning [53]. Compared with saplings, PC_sto_ and PC_NSC_ were associated with a broader suite of soil factors in young trees, potentially reflecting greater nutrient dependence during canopy expansion [15,54].

In adult trees, PC_eco_ was negatively associated with pH, whereas no other significant soil–trait associations were detected (Figure 2C1–C3). As rooting systems expand with tree size, adult trees may integrate water and nutrients over larger soil volumes, weakening the correspondence between leaf traits and locally measured surface-soil conditions [21,33]. The significant PC_eco_–pH association detected in adult trees may arise because soil pH integrates multiple aspects of nutrient availability and cycling, thereby representing a broad soil chemical gradient to which PC_eco_ remains responsive [20,55]. It should be noted that soil measurements were restricted to the 0–10 cm layer within a 1 m radius of each tree, which may not fully characterize the resource environment used by adult trees. Because local competition and microsite disturbance history were not included in the analysis, their potential influence on the stage-specific soil–trait associations cannot be excluded. The sample of 10 independent trees per stage may also have limited our ability to detect some associations. Future studies could combine larger samples and soil measurements across depths and distances with records of neighbouring trees, stand history, root distribution, and resource uptake across multiple stands.

## 5. Conclusions

In summary, our study shows that leaf functional variation in Fraxinus mandshurica is multidimensional and exhibits dimension-specific patterns across ontogenetic stages. Leaf economic, stomatal, and non-structural carbohydrate dimensions did not vary in parallel, and the dominant leaf functional dimension differed among ontogenetic stages, indicating that intraspecific functional variation extends beyond a single acquisitive–conservative continuum. Soil–trait associations also varied among ontogenetic stages and functional dimensions, with associations detected for a broader range of soil factors in saplings and young trees than in adult trees. Our findings show that ontogenetic stages differ across multiple leaf functional dimensions and in their associations with soil factors, suggesting that the ontogenetic stage and multiple functional dimensions should be considered together when evaluating intraspecific functional variations and soil–trait associations.

## Figures and Tables

**Table 1:** Summary of leaf functional traits across ontogenetic stages of *Fraxinus mandshurica*.

Ontogenetic Stage	Leaf Traits	Abbreviation	Units	Min	Max	Mean	SD	SE	CV (%)
Sapling	Specific leaf area	SLA	cm^2^ g^−1^	72.68	331.00	161.34 a	63.61	8.21	39.76
	Leaf dry matter content	LDMC	g g^−1^	0.16	0.45	0.31 c	0.05	0.006	15.36
	Leaf thickness	LT	mm	0.07	0.22	0.13 c	0.04	0.005	29.23
	Leaf nitrogen content	LN	mg g^−1^	30.27	58.53	43.15 a	6.27	0.81	14.66
	Stomatal length	SL	μm	11.64	20.59	16.84 b	1.80	0.23	10.79
	Stomatal width	SW	μm	7.39	18.92	13.72 b	2.06	0.26	15.00
	Stomatal density	SD	stoma mm^−2^	101.42	164.37	127.80 a	16.05	2.07	12.56
	Stomatal pore index	SPI	%	2.18	5.18	3.64 b	0.79	0.10	21.77
	Non-structural carbohydrates	NSC	% DM	7.85	19.74	12.01 b	2.72	0.35	22.68
	Leaf soluble sugars	LSS	% DM	2.07	6.80	4.41 c	1.08	0.14	24.60
	Leaf starch	LST	% DM	4.58	16.10	7.60 b	2.49	0.32	32.79
Young	Specific leaf area	SLA	cm^2^ g^−1^	63.09	191.31	113.30 b	31.03	4.01	27.39
	Leaf dry matter content	LDMC	g g^−1^	0.18	0.51	0.36 b	0.07	0.008	17.99
	Leaf thickness	LT	mm	0.08	0.24	0.17 a	0.04	0.005	22.89
	Leaf nitrogen content	LN	mg g^−1^	31.55	55.95	42.06 a	5.14	0.66	12.32
	Stomatal length	SL	μm	15.25	24.11	19.18 a	1.87	0.24	9.85
	Stomatal width	SW	μm	11.25	21.04	15.01 a	1.58	0.20	10.53
	Stomatal density	SD	stoma mm^−2^	83.94	181.86	122.80 a	19.99	2.58	16.28
	Stomatal pore index	SPI	%	2.44	7.16	4.58 a	1.20	0.16	26.51
	Non-structural carbohydrates	NSC	% DM	8.30	20.36	12.54 b	2.71	0.35	21.58
	Leaf soluble sugars	LSS	% DM	2.37	8.60	4.82 b	1.13	0.15	23.56
	Leaf starch	LST	% DM	4.31	15.48	7.73 b	2.31	0.30	29.91
Adult	Specific leaf area	SLA	cm^2^ g^−1^	61.16	166.23	106.55 b	23.04	2.99	21.80
	Leaf dry matter content	LDMC	g g^−1^	0.31	0.49	0.38 a	0.05	0.006	11.92
	Leaf thickness	LT	mm	0.07	0.22	0.16 b	0.04	0.005	23.81
	Leaf nitrogen content	LN	mg g^−1^	34.39	51.00	42.40 a	4.27	0.55	10.09
	Stomatal length	SL	μm	14.92	21.02	18.79 a	1.13	0.15	6.04
	Stomatal width	SW	μm	10.96	18.67	15.29 a	1.33	0.17	8.74
	Stomatal density	SD	stoma mm^−2^	87.43	153.88	122.44 a	15.99	2.06	13.06
	Stomatal pore index	SPI	%	3.36	6.75	4.31 a	0.58	0.07	13.53
	Non-structural carbohydrates	NSC	% DM	15.68	32.44	22.36 a	2.78	0.36	12.45
	Leaf soluble sugars	LSS	% DM	4.89	5.79	5.26 a	0.16	0.02	3.16
	Leaf starch	LST	% DM	10.53	27.00	17.10 a	2.75	0.35	16.20

Different lowercase letters following mean values indicate significant differences among ontogenetic stages at *p* < 0.05. Min, minimum; Max, maximum; SD, standard deviation; SE, standard error; CV, coefficient of variation.

**Table 2:** Principal component analysis of leaf functional traits in *Fraxinus mandshurica* across ontogenetic stages. Eigenvalues, explained variance, and trait loadings are shown for the first three principal components in saplings, young trees, adult trees, and all trees pooled. Bold values indicate traits with relatively strong contributions to the corresponding principal component.

	Sapling			Young Tree			Adult Tree			All Trees		
	PC1	PC2	PC3	PC1	PC2	PC3	PC1	PC2	PC3	PC1	PC2	PC3
Eigenvalue	3.25	2.04	1.85	3.69	2.23	1.66	2.73	2.14	1.62	3.85	1.86	1.55
Variation explained (%)	29.50%	18.50%	16.80%	33.60%	20.30%	15.10%	24.80%	19.40%	14.70%	35.00%	16.90%	14.10%
SLA	**0.41**	0.19	0.30	**0.44**	0.22	0.15	0.25	0.35	0.36	**0.40**	0.16	0.10
LDMC	−0.36	0.07	−0.16	−0.3	−0.002	−0.17	−0.39	0.21	−0.05	−0.37	−0.12	−0.21
LT	−0.35	−0.15	−0.28	−0.38	−0.19	−0.26	−0.11	−0.09	**−0.61**	−0.28	−0.33	−0.05
LN	0.36	−0.12	0.23	0.17	−0.18	0.08	0.14	0.17	**0.49**	0.20	0.245	0.23
SL	−0.27	−0.36	**0.46**	−0.28	0.14	**0.57**	**0.48**	0.11	−0.24	−0.35	−0.04	**0.52**
SW	−0.19	−0.31	0.37	−0.07	0.23	**0.55**	**0.42**	0.10	−0.18	−0.26	0.21	**0.51**
SD	−0.03	**0.50**	0.01	−0.33	−0.30	−0.08	−0.38	0.20	0.12	−0.007	**−0.42**	−0.33
SPI	−0.29	−0.06	**0.46**	−0.39	−0.09	0.384	0.08	0.31	−0.11	−0.31	0.31	0.29
NSC	−0.3	**0.45**	0.20	−0.19	**0.58**	−0.21	0.28	**−0.53**	0.13	−0.33	**0.47**	−0.26
LSS	−0.36	0.02	−0.23	−0.29	0.23	−0.09	−0.19	−0.29	0.33	−0.34	0.01	−0.20
LST	−0.17	**0.48**	0.32	−0.07	**0.57**	−0.20	0.29	**−0.52**	0.11	−0.29	**0.50**	−0.24

Trait loadings with absolute values ≥ 0.40 are shown in bold.

## Data Availability

The original contributions presented in this study are included in the article. Further inquiries can be directed to the corresponding author.

## Supplementary Materials

The following supporting information can be downloaded at: https://www.mdpi.com/article/10.3390/plants15203155/s1.

## Abbreviations

The following abbreviations are used in this manuscript:

SWC	Soil water content
pH	Soil pH
STC	Soil total carbon
STN	Soil total nitrogen
STP	Soil total phosphorus
