# Supplementary material for: Ontogenetic Variation in Multidimensional Leaf Traits and Stage-Specific Soil–Trait Associations in Fraxinus mandshurica
Source: Plants (Basel). 2026 Oct 15;15(20):3155. doi: 10.3390/plants15203155 (PMC13708691; doi:10.3390/plants15203155)
Supplement: Supplementary file 1 [file plants-15-03155-s001.zip › plants-4606911-supplementary.pdf]

Supplementary files

Figure S1. Three-dimensional PCA ordinations and two-dimensional projections of leaf functional traits in Fraxinus mandshurica across ontogenetic stages. (A1–A4) sapling; (B1–B4) young tree; (C1–C4) adult tree; and (D1–D4) all trees pooled. Arrows represent trait loadings, with arrow length indicating loading magnitude and arrow direction indicating the association of each trait with the principal component axes. Green, blue and red arrows represent leaf economic, stomatal and non-structural carbohydrate traits, respectively. Each point represents the mean trait values for a given leaflet position within an individual tree. Percentages in parentheses indicate the variance explained by each principal component. See Table 1 for trait abbreviations.

Figure S1

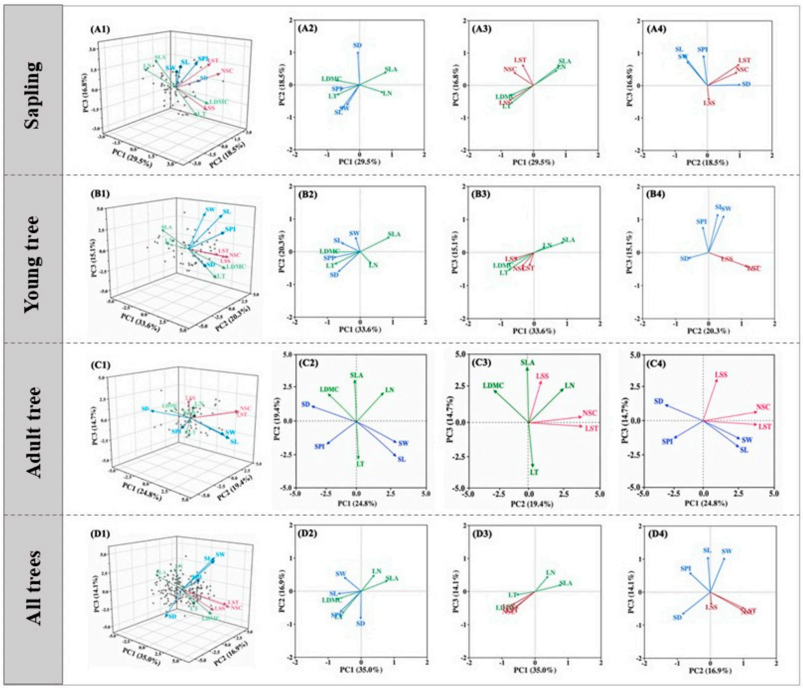

Figure S2. Principal component analysis of leaf economic, stomatal and non-structural carbohydrate traits of *Fraxinus mandshurica*, with data from all ontogenetic stages pooled. Points represent position-specific mean trait values for individual trees. Arrows represent trait loadings, with arrow length and direction indicating the magnitude and direction of each trait's association with the principal component axes. Percentages in parentheses indicate the variance explained by each principal component. See Table 1 for trait abbreviations.

Figure S2

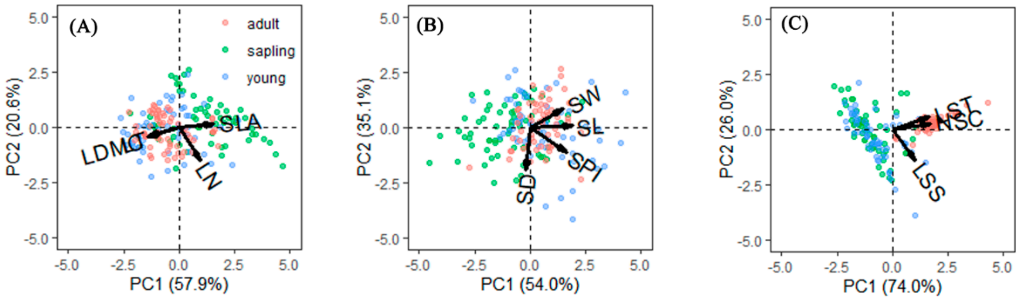

Table S1. Descriptive statistics of soil factors associated with *Fraxinus mandshurica* across ontogenetic stages. Different lowercase letters following mean values denote significant differences across ontogenetic stages at $p < 0.05$.

Ontogenetic stage	Soil factors	Abbreviation	Units	Min	Max	Mean	SD	SE	CV (%)
Sapling	Soil water content	SWC	g g^{-1}	0.33	0.79	0.55b	0.16	0.05	29.59
	Soil pH	pH	–	5.80	6.80	6.29a	0.31	0.10	5.00
	Soil total carbon	STC	mg g^{-1}	40.04	117.38	80.30b	31.07	9.83	38.69
	Soil total nitrogen	STN	mg g^{-1}	3.90	10.17	6.94c	2.22	0.70	31.69
	Soil total phosphorus	STP	mg g^{-1}	0.67	1.67	1.01b	0.33	0.10	32.50
Young	Soil water content	SWC	g g^{-1}	0.41	0.75	0.52b	0.10	0.03	19.49
	Soil pH	pH	–	5.70	6.78	6.10b	0.35	0.11	5.70
	Soil total carbon	STC	mg g^{-1}	45.11	142.66	97.32a	28.86	9.13	29.66
	Soil total nitrogen	STN	mg g^{-1}	3.86	10.15	7.86b	2.07	0.66	26.37
	Soil total phosphorus	STP	mg g^{-1}	0.56	1.45	1.05b	0.28	0.09	26.29
Adult	Soil water content	SWC	g g^{-1}	0.43	0.79	0.62a	0.13	0.04	20.27
	Soil pH	pH	–	5.21	6.64	6.08b	0.52	0.16	8.52
	Soil total carbon	STC	mg g^{-1}	76.69	136.79	105.46a	17.62	5.57	16.71
	Soil total nitrogen	STN	mg g^{-1}	6.50	14.21	8.83a	2.25	0.71	25.49
	Soil total phosphorus	STP	mg g^{-1}	0.92	2.10	1.31a	0.44	0.14	33.53

SWC, soil water content; STC, soil total carbon; STN, soil total nitrogen; STP, soil total phosphorus.

Table S2. Results of likelihood ratio tests evaluating the effects of ontogenetic stage and leaflet position on leaf functional trait axes.

Response variable	Effect	χ^2	df	*p*
PC_{eco}	Ontogenetic stage	24.8	2	<0.001
	Leaflet position	138.88	5	<0.001
	Stage × Position	15.09	10	0.129
PC_{sto}	Ontogenetic stage	22.88	2	<0.001
	Leaflet position	7.49	5	0.187
	Stage × Position	2.46	10	0.991
PC_{NSC}	Ontogenetic stage	79.3	2	<0.001
	Leaflet position	18.8	5	0.002
	Stage × Position	12.14	10	0.276

Table S3. Standardized regression coefficients from stage-specific multiple linear models relating soil factors to the leaf economic (PC_{eco}), stomatal (PC_{sto}) and non-structural carbohydrate (PC_{NSC}) axes of *Fraxinus mandshurica*. Models were fitted using position-specific PC scores, with each tree's soil measurements matched to its six leaflet-position observations.

	Sapling				Young tree				Adult tree			
	Soil factors	β	95% CI	*p*	Soil factors	β	95% CI	*p*	Soil factors	β	95% CI	*p*
PC_{eco}	SWC	-0.295	-0.550, -0.040	0.023*	SWC	-0.253	-0.694, 0.188	0.261	SWC	0.302	-0.370, 0.974	0.379
	pH	0.205	-0.058, 0.469	0.126	pH	0.628	0.339, 0.917	<0.001***	pH	-1.003	-1.751, -0.256	0.008**
	STN	1.398	0.591, 2.205	<0.001***	STN	-1.193	-2.036, -0.350	0.006**	STN	2.366	-0.017, 4.750	0.052
	STP	0.784	0.264, 1.305	0.003**	STP	0.904	0.541, 1.267	<0.001***	STP	-1.408	-2.990, 0.173	0.081
	STC	-1.476	-2.209, -0.743	<0.001***	STC	1.11	0.444, 1.776	0.001**	STC	-0.967	-2.139, 0.206	0.106
PC_{sto}	SWC	0.478	0.130, 0.826	0.007**	SWC	-0.421	-0.840, -0.003	0.048*	SWC	-0.113	-0.572, 0.346	0.63
	pH	0.174	-0.186, 0.534	0.343	pH	-0.233	-0.507, 0.041	0.096	pH	-0.331	-0.841, 0.179	0.204
	STN	-0.413	-1.515, 0.689	0.463	STN	0.634	-0.166, 1.433	0.12	STN	1.031	-0.596, 2.659	0.214
	STP	0.308	-0.403, 1.018	0.396	STP	-0.615	-0.960, -0.271	<0.001***	STP	-0.6	-1.679, 0.480	0.277
	STC	-0.079	-1.079, 0.921	0.877	STC	0.087	-0.545, 0.718	0.788	STC	-0.356	-1.157, 0.445	0.383
PC_{NSC}	SWC	0.411	0.096, 0.726	0.011*	SWC	-0.215	-0.579, 0.150	0.248	SWC	0.274	-0.129, 0.677	0.183
	pH	0.115	-0.211, 0.441	0.49	pH	-0.251	-0.489, -0.012	0.039*	pH	-0.107	-0.555, 0.341	0.64
	STN	-0.343	-1.342, 0.656	0.501	STN	-0.511	-1.207, 0.185	0.15	STN	0.761	-0.669, 2.191	0.297
	STP	0.175	-0.469, 0.820	0.594	STP	-0.359	-0.659, -0.059	0.019*	STP	-0.732	-1.681, 0.217	0.131
	STC	-0.294	-1.201, 0.613	0.525	STC	0.676	0.126, 1.226	0.016*	STC	-0.111	-0.814, 0.593	0.758
